# A phase II trial to assess efficacy and safety of afatinib in extensively pretreated patients with HER2-negative metastatic breast cancer

**DOI:** 10.1007/s10549-012-2126-1

**Published:** 2012-07-05

**Authors:** Martin Schuler, Ahmad Awada, Philipp Harter, Jean Luc Canon, Kurt Possinger, Marcus Schmidt, Jacques De Grève, Patrick Neven, Luc Dirix, Walter Jonat, Matthias W. Beckmann, Jochen Schütte, Peter A. Fasching, Nina Gottschalk, Tatiana Besse-Hammer, Frank Fleischer, Sven Wind, Martina Uttenreuther-Fischer, Martine Piccart, Nadia Harbeck

**Affiliations:** 1 Department of Medical Oncology, West German Cancer Center, University Hospital Essen, University of Duisburg-Essen, Essen, Germany; 2 Institut Jules Bordet, Université Libre de Bruxelles, Ixelles, Belgium; 3 HSK, Dr Horst Schmidt Kliniken GmbH, Wiesbaden, Germany; 4 Department of Medical Oncology, Grand Hopital de Charleroi, Charleroi, Belgium; 5 Department of Hematology and Oncology, Charité, Berlin, Germany; 6 Department of Obstetrics and Gynecology, University Hospital, Mainz, Germany; 7 Oncologisch Centrum, UZBrussel, Brussels, Belgium; 8 Multidisciplinary Breast Centre Leuven University Hospital, Leuven, Belgium; 9 Oncology Centre, General Hospital St-Augustinus, Antwerp, Belgium; 10 Department of Obstetrics and Gynecology, University of Kiel, Kiel, Germany; 11 Department of Obstetrics and Gynecology, University Breast Center Franconia, Comprehensive Cancer Center Erlangen-Nuremberg, University Hospital Erlangen, Friedrich-Alexander University Erlangen-Nuremberg, Erlangen, Germany; 12 Department of Oncology, Marien Hospital, Düsseldorf, Germany; 13 Department of Obstetrics and Gynecology, Technische Universität München, Munich, Germany; 14 Boehringer Ingelheim GmbH & Co. KG, Biberach, Germany; 15 Department of Obstetrics and Gynecology, Breast Center, University of Munich, Klinikum Grosshadern, Marchioninistr. 15, 81377 Munich, Germany

**Keywords:** Afatinib, Metastatic breast cancer, Triple-negative breast cancer, HER2-negative breast cancer, EGFR TKI

## Abstract

Afatinib (BIBW 2992) is an ErbB-family blocker that irreversibly inhibits signaling from all relevant ErbB-family dimers. Afatinib has demonstrated preclinical activity in human epidermal growth factor receptor HER2 (ErbB2)-positive and triple-negative xenograft models of breast cancer, and clinical activity in phase I studies. This was a multicenter phase II study enrolling patients with HER2-negative metastatic breast cancer progressing following no more than three lines of chemotherapy. No prior epidermal growth factor receptor-targeted therapy was allowed. Patients received 50-mg afatinib once daily until disease progression. Tumor assessment was performed at every other 28-day treatment course. The primary endpoint was clinical benefit (CB) for ≥4 treatment courses in triple-negative (Cohort A) metastatic breast cancer (TNBC) and objective responses measured by Response Evaluation Criteria in Solid Tumors in patients with HER2-negative, estrogen receptor-positive, and/or progesterone receptor-positive breast cancer (Cohort B). Fifty patients received treatment, including 29 patients in Cohort A and 21 patients in Cohort B. No objective responses were observed in either cohort. Median progression-free survival was 7.4 and 7.7 weeks in Cohorts A and B, respectively. Three patients with TNBC had stable disease for ≥4 treatment courses, one of them for 12 courses (median 26.3 weeks; range 18.9–47.9 weeks). The most frequently observed afatinib-associated adverse events (AEs) were gastrointestinal and skin-related side effects, which were manageable by symptomatic treatment and dose reductions. Afatinib pharmacokinetics were comparable to those observed in previously reported phase I trials. In conclusion, afatinib had limited activity in HER2-negative breast cancer. AEs were generally manageable and mainly affected the skin and the gastrointestinal tract.

**Electronic supplementary material:**

The online version of this article (doi:10.1007/s10549-012-2126-1) contains supplementary material, which is available to authorized users.

## Introduction

Breast cancer is the leading single cause of cancer-related mortality in women worldwide, accounting for more than 450,000 deaths in 2008 (14 % of cancer deaths) [1]. Breast cancer is not a single disease, but a collection of subtypes based on genotyping and biological properties [2]. Hormone receptors, HER2 expression, and genomic profiling distinguish four major expression profile subtypes with clinical significance: luminal subtype A, luminal subtype B, HER2-positive tumors, and basal-like tumors [3–5].

The triple-negative breast cancers (TNBC)—so called because they are negative for estrogen receptor (ER), progesterone receptor (PgR), and HER2 [6]—include a large proportion of basal-like tumors. About 15 % of breast cancers are TNBC and, despite being sensitive to systemic chemotherapies, these tumors are, overall, associated with poor outcome compared with other subtypes of breast cancer, with a recurrence rate of 30–40 % presenting as distant metastases [7]. Apart from chemotherapy, TNBC patients have few treatment options as these tumors do not overexpress antigens that would allow for targeted treatment, such as endocrine and anti-HER2 therapy. Overexpression of epidermal growth factor receptor (EGFR/ErbB1) has been reported in TNBC [8–11] and may therefore be a valid target for anti-tumor therapy in TNBC. A phase II study of the anti-EGFR monoclonal antibody, cetuximab, in combination with cisplatin, demonstrated a modest improvement in outcome compared with cisplatin alone [12]. EGFR mutations have also been reported in 11 % of TNBC patients [11]. These findings suggest that EGFR may play a role in the development of metastases [13] and thus could become a relevant target in TNBC. While patients with HER2-negative, hormone receptor (HR)-positive breast cancer are often responsive to endocrine therapy at first presentation, acquired resistance leading to relapse, disease progression, and death is common [14].

Afatinib (BIBW 2992) is a novel, potent, orally bioavailable, ErbB-family blocker which irreversibly inhibits all ErbB-family members with intrinsic catalytic activity, including EGFR (maximal inhibitory concentration [IC_50_] 0.5 nM), HER2 (IC_50_ 14 nM) [15], and HER4 (ErbB4) (IC_50_ 1 nM) receptors (Dahl et al., manuscript submitted). Afatinib also inhibits HER3 (ErbB3) transphosphorylation [15]. In vitro, afatinib has demonstrated anti-proliferative activity in HER2-positive and in “triple negative” breast cancer cell lines, including the EGFR-expressing SUM 190 and SUM-149 cell lines [16, 17]. Anti-tumor activity of afatinib has also been confirmed in vivo in mice carrying HER2-amplified or TNBC xenografts [17].

The rationale for studying afatinib in the treatment of non-HER2-amplified breast cancer was based on several factors. First, the high expression of EGFR in some TNBC patients [18] and the assumption that uncontrolled ErbB-signaling is directly related to an increased oncogenic potential in TNBC subtypes. Second, the transcriptional repressor activity of the ER on ErbB-family members [19–21] suggests that in ER-positive patients, abating the natural activation of ER signaling by its ligand estradiol may bring about the use of alternative proliferation pathways, including the ErbB-signaling network. A rational treatment approach in HER2-negative, HR-positive breast cancer patients who have progressed on endocrine treatment may therefore benefit from an agent such as afatinib.

This study was undertaken to assess the efficacy, safety, and pharmacokinetics (PK) of afatinib monotherapy in patients with HER2-negative metastatic breast cancer—i.e., patients with TNBC (Cohort A) and patients with HER2-negative, HR-positive disease (Cohort B).

## Patients and methods

### Study population

Female patients aged ≥18 years with HER2-, ER-, and PgR-negative TNBC (Cohort A) and HER2-negative, ER-, and/or PgR-positive breast cancer (Cohort B) were enrolled based on documented HER2/ER and PgR-status. All patients were required to have histologically proven metastatic (disease stage IV) breast cancer, measurable disease, failed or relapsed after no more than two (Cohort B) or three (Cohort A as per protocol amendment) lines of chemotherapy, including adjuvant therapy. Patients were also required to have Eastern Cooperative Oncology Group (ECOG) performance status of 0–2 and had to have archival tumor tissue available. HER2-positive status was assessed using immunohistochemistry (IHC) with use of fluorescence in situ hybridization (FISH) as a confirmatory test in patients whose samples were HER2 2+ by IHC. ER and PgR-status were to be assessed by IHC in Cohorts A and B, with an Allred Score 2/8 or below confirming HR-negative and a score of ≥3/8 HR-positive status [22].

Patients were to be excluded for active infectious disease; gastrointestinal disorders that may interfere with the absorption of the study drug or chronic diarrhea; active/symptomatic brain metastases; cardiac left ventricular function with resting ejection fraction <50 %; absolute neutrophil count <1,500 cells/mm^3^; platelet count <100,000 cells/mm^3^; bilirubin >1.5 mg/dL (>26 μmol/L, SI equivalent) and serum creatinine >1.5 mg/dL (>132 μmol/L, SI unit equivalent); and previous treatment with trastuzumab or EGFR/HER2 inhibitors. Co-medication with corticosteroids and bisphosphonates was permitted.

### Study design

This was an open-label, multicenter (13 centers in Germany and Belgium), phase II study of afatinib in two cohorts of patients with HER2-negative metastatic breast cancer. Forty patients were planned to be treated per cohort, following a Gehan two-stage design [23]. The study would be terminated early if no benefit was observed in the initial 20 patients treated per cohort, i.e., in Cohort A (TNBC) ≥3 patients with CB in 20 patients and in Cohort B (HER2-negative/ER- and/or PgR-positive) no objective response in 20 patients.

After a 14-day screening period, patients started trial treatment with afatinib. Planned visits were scheduled for Days 1 and 14 during the first two courses, thereafter only on Day 1 of each course. An end-of-trial visit was carried out at the end of treatment, with a follow-up visit 28 ± 7 days later.

The study was conducted in accordance with the Declaration of Helsinki, local laws and the International Conference on Harmonization—Good Clinical Practice Guideline, and approved by all relevant regulatory and independent ethics committees or institutional review boards. All patients provided written informed consent prior to inclusion in the study.

### Study treatment

Afatinib (Boehringer Ingelheim Pharma GmbH & Co. KG) 50 mg once daily was administered orally as film-coated tablets of 20- and 5-mg tablet strength. Patients received continuous daily dosing from Day 1 (visit 1). Each course consisted of 28 days. Treatment continued until disease progression, unacceptable adverse events (AEs) and non-compliance, or withdrawal of consent. Dose reductions and transient interruptions of treatment (up to 14 days) were permitted to manage AEs and additional guidance for the management of diarrhea and skin rashes associated with EGFR inhibitors was incorporated into the trial protocol during study conduct.

### Efficacy assessments

Response evaluation was performed according to Response Evaluation Criteria in Solid Tumors (RECIST) 1.0 criteria. The primary endpoint was objective response [partial response (PR) + complete response (CR)] in Cohort B. In Cohort A, the primary endpoint was changed from objective response to CB [CR + PR + stable disease (SD) for ≥4 months] with a protocol amendment. Assessments were performed as close as possible for 8 weeks, but no earlier than 4 weeks after the start of treatment. An end-of-trial assessment was to be done, unless end-of-trial coincided with a scheduled visit.

Secondary efficacy endpoints included: objective response for Cohort A and CB for Cohort B, time to and duration of objective response; time to tumor progression; progression-free survival (PFS); overall survival (OS); safety, including changes in left ventricular ejection fraction and PK.

### Biomarkers

Tumor samples from both patient Cohorts were analyzed, or re-analyzed, for EGFR, HER2, PgR, ER status, and cytokeratin (CK) 5, 6, and 14. In addition, tumor tissue from patients in Cohort A, who agreed to fresh tumor biopsies prior to trial enrollment, was analyzed for EGFR and EGFR-ligand overexpression. Analysis of soluble HER2/neu extracellular domain (ECD), EGFR ECD, and tumor marker CA15.3 was also undertaken. Further details of biomarker assessments are given as supplementary information.

### Safety assessments

Patients were monitored for AEs during and after treatment. AEs were graded according to the National Cancer Institute Common Terminology Criteria for Adverse Events (NCI CTCAE) Version 3.0.

### Pharmacokinetic sampling and data analysis

Plasma concentrations of afatinib were quantified by a validated high-performance liquid chromatography tandem mass spectrometry method at Boehringer Ingelheim Pharma GmbH & Co. KG, Germany. Analyses were performed on 5 mL of venous blood collected prior to drug administration on Days 1 and 14 of treatment Courses 1 and 2, and approximately 1, 2, and 3 h after drug administration on Day 1/Course 1 and Day 14/Course 2. PK sampling was done prior to drug administration on Day 1 of subsequent courses.

### Statistical methods

Safety and efficacy parameters were evaluated descriptively. Kaplan–Meier estimates were used for analysis of PFS. Analyses were conducted to assess the prognostic effect of baseline characteristics on response using Cox proportional hazard models, by cohort. As the trial was not powered to show a statistically significant effect for any of the comparisons these analyses were considered exploratory.

## Results

### Patient population

A total of 50 patients received treatment with afatinib. Following the predefined Gehan stopping rule Cohort B was closed after 21 patients had been entered and no objective response was observed. Cohort A was intended to continue recruitment to full accrual of 40 patients, but was closed early due to slow recruitment. Patient demographics and baseline characteristics are given in Table 1. The majority of patients had received prior chemotherapy, only four patients had received previous neoadjuvant chemotherapy, radiotherapy, or surgical intervention; in addition, all patients in Cohort B had received prior anti-hormonal therapy. In the total population, the majority of patients (62.0 %) discontinued treatment due to disease progression.

**Table 1:** Patient demographics and baseline characteristics (treated set)

	Cohort A	Cohort B
Total treated, *n* (%)	29 (100)	21 (100)
Female, *n* (%)	29 (100)	21 (100)
Race, *n* (%)		
Black	0	1 (4.8)
White	29 (100)	20 (95.2)
Age (years)		
Median (range)	53.0 (33–75)	61.0 (39–87)
Weight (kg)		
Median (range)	70 (46–100)	67 (50–114)
Time since first-histological diagnosis (years)		
Median (range)	1.9 (0.1–17.2)	6.3 (1.2–32.2)
Number of metastatic sites		
Median (range)	2 (1–6)	2 (1–5)
Sites of metastases, *n* (%)		
Liver	12 (41.4)	13 (61.9)
Lung	10 (34.5)	13 (61.9)
Peritoneum	0	1 (4.8)
Brain	1 (3.4)	1 (4.8)
Other	26 (89.7)	16 (76.2)
Classification of primary tumor at diagnosis (%)		
ER status		
Positive	1 (3.4)	21 (100)
Negative	28 (96.6)	0
PgR-status		
Positive	1^a^ (3.4)	19 (90.5)
Negative	28 (96.6)	2 (9.5)
HER2 status		
Positive	1^a^ (3.4)	0
Negative	28 (96.6)	21 (100)
Type of previous therapies, *n* (%)		
Surgery	27 (93.1)	18 (85.7)
Chemotherapy	28 (96.6)	17 (81.0)
Radiotherapy	19 (65.5)	18 (85.7)
Hormone therapy	7 (24.1)	21 (100)
Immunological therapy	3 (10.3)	0
Number of prior chemotherapies, *n* (%)		
0	1 (3.4)	4 (19.0)
Neoadjuvant only	3 (10.3)	1 (4.8)
1–2	23 (79.3)	16 (76.2)
3	2 (6.9)	0

*ER* estrogen receptor, *HER2* human epidermal growth factor 2, *PgR* progesterone receptor

^a^One patient had HER2-positive, ER-positive, and PgR-positive breast cancer at study entry and was considered to be a protocol violation. However, a second biopsy performed on newly developed metastases showed that the patient had TNBC, thus the patient was included in all analyses

### Efficacy

**Fig. 1:**
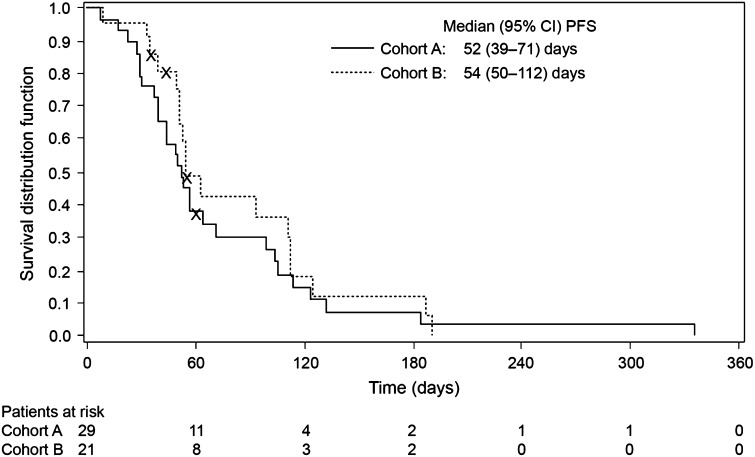
Kaplan–Meier curves for progression-free survival (treated set)

Patients’ response to treatment with afatinib is given in Table 2. No objective responses (CR + PR) were observed. Three patients in Cohort A and one patient in Cohort B had CB for a minimum of 4 months. In Cohort A, 28 (97 %) patients had disease progression and the median PFS was 7.4 weeks [95 % confidence interval (CI) 5.6–10.1 weeks). For three patients in Cohort A, who experienced CB, the median duration of PFS was 26.3 (range 18.9–47.9) weeks. Median PFS in Cohort B was 7.7 weeks (95 % CI 7.1–16.0 weeks). Figure 1 shows Kaplan–Meier curves for PFS. The median OS in Cohort A was not reached, whereas in Cohort B, the median OS was 64.0 weeks (95 % CI 44.3–76.7 weeks).

**Table 2:** Overview of response (according to RECIST evaluation)

	Cohort A *N* = 29 *n* (%)	Cohort B *N* = 21 *n* (%)
Number of patients with evaluation after baseline	27 (93.1)	18 (85.7)
Confirmed objective response	0	0
Confirmed best overall response		
Complete response	0	0
Partial response	0	0
Stable disease	7^b^ (24.1)	5^c^ (23.8)
Clinical benefit^a^	7^b^ (24.1)	5^c^ (23.8)
Modified clinical benefit (stable disease be observed ≥4 months after the start of treatment)	3^b^ (10.3)	1 (4.8)

*RECIST* Response Evaluation Criteria in Solid Tumors

^a^Defined as best overall response of complete response, partial response or stable disease (the latter is confirmed if the time point of measurement is ≥6 weeks (42 days) after administration

^b^Included one patient with HER2 IHC 2+ for whom FISH analysis was not evaluable, one patient with HER2 IHC 0, and one patient with a HER2 IHC 2+/FISH positive primary tumor who was included in the trial as a biopsy performed on metastases showed that the patient had TNBC

^c^Included one patient with HER2 IHC 2+ later confirmed HER2-positive by FISH

For patients in Cohort A, at the end of treatment ECOG performance score had improved in 1 (3.4 %) patient, remained unchanged in 12 (41.4 %) patients, and deteriorated in 15 (51.7 %) patients. In Cohort B, at the end of treatment the ECOG performance score had not improved in any patients, remained unchanged in 6 (28.6 %) patients, and deteriorated in 13 (61.9 %) patients.

### Biomarkers and exploratory analyses

Biomarker assessment on archival tissue biopsies collected upon trial entry confirmed HER2-negative status in 26/29 patients in Cohort A (Table 3); of the eight patients tested by FISH, two were not evaluable, one tested positive and five were confirmed negative. In Cohort B, 20/21 patients were confirmed HER2-negative by IHC and/or FISH; one HER2 2+ patient by IHC was confirmed HER2-positive by FISH (Table 3).

**Table 3:** Biomarker assessment at baseline

*n* (%)	Cohort A *N* = 29	Cohort B *N* = 21
PgR-status IHC		
Missing	1 (3.4)	1 (4.8)
Positive	3 (10.3)	12 (57.1)
Negative	25 (86.2)	8 (38.1)
PgR total score (Allred)		
Missing	2 (6.9)	1 (4.8)
≥3/8 (positive)	3 (10.3)	13 (61.9)
2/8 or below (negative)	24 (82.8)	7 (33.3)
ER status IHC		
Missing	1 (3.4)	1 (4.8)
Positive	3 (10.3)	19 (90.5)
Negative	25 (86.2)	1 (4.8)
ER total score (Allred)		
Missing	3 (10.3)	1 (4.8)
≥3/8 (positive)	3 (10.3)	19 (90.5)
2/8 or below (negative)	23 (79.3)	1 (4.8)
HER2 IHC		
Missing	0 (0.0)	0 (0.0)
0	15 (51.7)	10 (47.6)
1+	10 (34.5)	4 (19.0)
2+	4 (13.8)	7 (33.3)
3+	0 (0.0)	0 (0.0)
HER2 FISH		
Positive	1 (3.4)	1 (4.8)
Negative	5 (17.2)	6 (28.6)
Not evaluable	2 (6.9)	0 (0.0)
Not applicable	21 (72.4)	14 (66.7)
EGFR IHC		
Missing	1 (3.4)	1 (4.8)
Positive	18 (62.1)	2 (9.5)
Negative	9 (31.0)	17 (81.0)
Not evaluable	1 (3.4)	1 (4.8)
EGFR FISH^a^		
Missing	12 (41.4)	7 (33.3)
Positive	1 (3.4)	0 (0.0)
Negative	0 (0.0)	0 (0.0)
Not evaluable	16 (55.2)	14 (66.7)
Cytokeratin 5 test		
Missing	13 (44.8)	7 (33.3)
Positive	0 (0.0)	0 (0.0)
Negative	0 (0.0)	0 (0.0)
Not evaluable	16 (55.2)	14 (66.7)
Cytokeratin 5/6 ratio test		
Missing	1 (3.4)	1 (4.8)
Positive	17 (58.6)	0 (0.0)
Negative	10 (34.5)	20 (95.2)
Not evaluable	1 (3.4)	0 (0.0)
Cytokeratin 5/14 ratio test		
Missing	3 (10.3)	1 (4.8)
Positive	16 (55.2)	1 (4.8)
Negative	9 (31.0)	19 (90.5)
Not evaluable	1 (3.4)	0 (0.0)

*EGFR* epidermal growth factor receptor, *FISH* fluorescence in situ hybridization, *HER2* human epidermal growth factor 2, *IHC* immunohistochemistry

^a^EGFR FISH was only performed in cases where EGFR IHC was positive

Changes in serum levels of CA 15.3, HER2 ECD and EGFR ECD over the course of the study showed no conclusive trends.

**Fig. 2:**
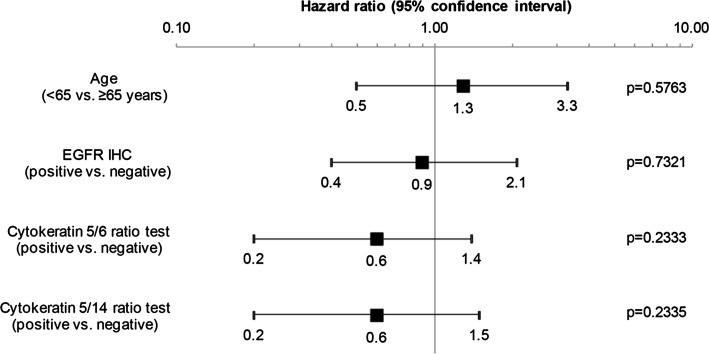
Hazard ratio (95 % CI) of progression-free survival by baseline characteristics for Cohort A. *EGFR* epidermal growth factor receptor, *IHC* immunohistochemistry

Hazard ratio (95 % CI) of PFS by baseline characteristics for Cohort A is shown in Fig. 2. For Cohort B, patient numbers were inadequate for the analysis for any subgroups except for age. Age was associated with a small magnitude of effect on PFS in both Cohort A (Fig. 2) and Cohort B (hazard ratio 1.1, 95 % CI 0.4, 2.9, *p* = 0.8585).

### Safety and tolerability

The frequency of treatment-related AEs, including CTCAE grade ≥3, across both the study cohorts is summarized in Table 4. The majority (96.0 %) of patients had at least one drug-related AE during the study. Fifteen (51.7 %) patients in Cohort A and 15 (71.4 %) patients in Cohort B had AEs leading to dose reduction. Diarrhea was the most common cause of dose reductions [Cohort A: 13 (44.8 %) patients; Cohort B: 9 (42.9 %) patients]. Twenty-three (46.0 %) patients discontinued permanently from the study due to drug-related AEs, mainly diarrhea (24.0 %).

**Table 4:** Treatment-related adverse events occurring in >10 % of patients in either cohort; adverse events reported as NCI CTCAE grades

	Cohort A *N* = 29 *n* (%)		Cohort B *N* = 21 *n* (%)		Total *N* = 50 *n* (%)	
	All grades	≥Grade 3	All grades	≥Grade 3	All grades	≥Grade 3
Patient with any treatment-related adverse event	28 (96.6)	19 (65.6)	20 (95.2)	13 (61.9)	48 (96.0)	32 (64.0)
Adverse event						
Diarrhea	28 (96.6)	14 (48.3)	18 (85.7)	6 (28.6)	46 (92.0)	20 (40.0)
Rash	9 (31.0)	1 (3.4)	9 (42.9)	1 (4.8)	18 (36.0)	2 (4.0)
Nausea	9 (31.0)	2 (6.9)	7 (33.3)	3 (14.3)	16 (32.0)	5 (10.0)
Mucosal inflammation	8 (27.6)	2 (6.9)	6 (28.6)	1 (4.8)	14 (28.0)	3 (6.0)
Acne	10 (43.5)	1 (3.4)	3 (14.3)	0	13 (26.0)	1 (2.0)
Fatigue	7 (24.1)	2 (6.9)	5 (23.8)	3 (14.3)	12 (24.0)	5 (10.0)
Dry skin	8 (27.6)	0	4 (19.0)	0	12 (24.0)	0
Decreased appetite	5 (17.2)	1 (3.4)	6 (28.6)	0	11 (22.0)	1 (2.0)
Vomiting	5 (17.2)	0	6 (28.6)	1 (4.8)	11 (22.0)	1 (2.0)
Stomatitis	5 (17.2)	1 (3.4)	2 (9.5)	0	7 (14.0)	1 (2.0)
Epistaxis	3 (10.3)	0	3 (14.3)	0	6 (12.0)	0
Palmar–plantar erythrodysesthesia syndrome	1 (3.4)	0	4 (19.0)	1 (4.8)	5 (10.0)	1 (2.0)
Skin fissures	2 (6.9)	0	3 (14.3)	0	5 (10.0)	0
Dermatitis acneiform	1 (3.4)	0	3 (14.3)	1 (4.8)	4 (8.0)	1 (2.0)
Abdominal pain	3 (10.3)	0	1 (4.8)	0	4 (8.0)	0
Dyspnea	0	0	3 (14.3)	1 (4.8)	3 (6.0)	1 (2.0)

*NCI CTCAE* National Cancer Institute Common Terminology Criteria for Adverse Events (version 3)

Serious AEs (SAEs) occurred in 20 (40.0 %) patients; 13 (44.8 %) patients in Cohort A and 7 (33.3 %) in Cohort B. Five fatal events were reported in 4 (13.8 %) patients in Cohort A and 1 (4.8 %) patient in Cohort B. One of these was considered to be drug-related; the patient died from bronchopneumonia and reduced general state, the latter being related to diarrhea. Another patient developed acute renal failure 29 days after study medication had been discontinued and subsequently died in the post-study period. Study medication had been discontinued due to progressive disease and 2 days after the start of doxorubicin treatment the patient developed renal failure.

Three patients showed a drug-related reduction in cardiac left ventricular ejection fraction. One patient reported CTCAE grade 1 ventricular failure with moderate aortic stenosis and hypertension on treatment, one patient reported post-treatment CTCAE grade 2 decrease of ejection fraction and one patient suffered from hypertension, obstructive pulmonary disease and was found to have a pulmonary embolus post-study with no AE reported. No treatment was required in these patients.

### Pharmacokinetics

**Fig. 3:**
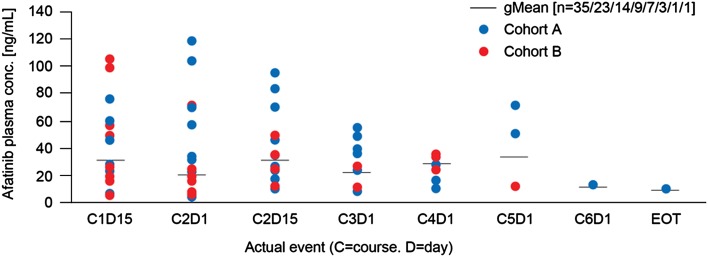
Predose afatinib plasma concentrations at steady state (50 mg). *HER* human epidermal growth factor receptor, *HR* hormone receptor

No differences in plasma concentrations were detected between the Cohorts (Fig. 3). In both the cohorts, afatinib plasma levels had reached steady state by Day 14 at the latest. Steady state may have been reached earlier, but this could not be corroborated because PK sampling was not done between Days 1 and 14.

## Discussion

This was an open-label, phase II study designed to evaluate the efficacy and safety of once daily oral treatment with afatinib in patients with HER2-negative metastatic breast cancer after failure of prior chemotherapy regimens. Afatinib displayed modest anti-tumor activity in patients with TNBC. Three (10.3 %) patients with TNBC in Cohort A and one (4.8 %) patient with HER2-negative, HR-positive metastatic breast cancer in Cohort B showed prolonged CB with SD for at least 4 months after the start of treatment. No objective responses (primary endpoint for Cohort B) were observed in this study. Although the median PFS was low in both the cohorts (~7 weeks), it is notable that the median PFS was markedly prolonged in the three patients in Cohort A who had continued CB for more than 4 months (median 26.3 weeks with one patient having a PFS of 47.9 weeks), clearly indicating therapeutic benefit in these patients. Interestingly, PFS in Cohorts A and B was similar, despite the poor prognosis and OS of patients with metastatic TNBC [24].

With regards to retrospective exploratory biomarker analyses, the main prognostic factor to have an effect on PFS in Cohort A was intratumoral expression of CK; patients with a negative result at baseline generally had longer PFS compared to those with a positive baseline result. This should be considered hypothesis-generating due to the small numbers of patients and the fact that the trial was not powered to show a statistically significant effect for these comparisons. However, these findings would perhaps be expected given that expression of basal markers, such as CK, are related to a worse prognosis and identifies a clinically distinct subgroup of patients with TNBC [25].

The safety profile of afatinib was similar to that observed in previous studies in patients with other tumor types [26]; common AEs were related to skin and gastrointestinal tract. Side effects were manageable through treatment of symptoms and dose reduction. Diarrhea was reported in almost all the patients in both the cohorts and was the primary reason for dose interruption or reduction. The side-effect profile observed in this trial is consistent with the profile observed for other agents which target the HER family, particularly those that target EGFR tyrosine kinase inhibitors (TKIs), potentially because EGFR is largely expressed in cells of epithelial origin, such as those of the skin and the gastrointestinal tract [27].

At a starting does of afatinib 50 mg/day, the rate of discontinuation from the study because of AEs—particularly diarrhea—was higher than what was previously reported with reversible EGFR and EGFR/HER2 inhibitors in patients with breast cancer and may have contributed to the limited efficacy observed [28, 29]. This may have been a result of delayed management of side effects due to a lack of experience with this class of compounds. Furthermore, it may reflect a higher biologic activity of afatinib, an irreversible ErbB-family blocker, compared with other reversible agents that inhibit only one or two members of the HER family. Early and proactive management of afatinib-related AEs, including early start of anti-diarrheal therapy, dose reductions using an afatinib-specific dose reduction scheme and introduction of a lower starting dose of 40 mg, as recommended in phase II and phase III monotherapy studies, may increase tolerability and limit early treatment discontinuations due to AEs.

TNBC is a cancer with a high level of tumor heterogeneity, for which there are currently limited treatment options. While extended CB was observed in several patients in Cohort A, inclusion was based on HER2 status at trial entry with subsequent HER2 testing based on archived biopsies tissue. As tumors are heterogeneous, and changes in mutation status can occur, it might be possible that some patients who were classified as TNBC at study entry and who subsequently experienced CB during treatment with afatinib may, in fact, have had HER2-positive tumor sites at the time of study treatment. Further research is also needed to clarify the role of EGFR inhibition in the treatment of TNBC as preclinical research with reversible EGFR TKIs suggests a role for this treatment approach [7–10, 18, 30, 31]. A preclinical study of cetuximab and cisplatin in gefitinib-resistant TNBC cell lines demonstrated a potential benefit for this combination, although it also raised questions regarding the optimal treatment schedule and dosing for this combination, as cisplatin was able to deplete EGFR, the target of cetuximab [32].

Despite promising preclinical results, clinical trials conducted to date with EGFR-targeted therapies have shown mixed results in TNBC. In general, monotherapy with EGFR-targeted therapies have been associated with poorer outcomes than combinations with chemotherapy. The reversible EGFR/HER2 TKI, lapatinib, was associated with no benefit in patients with TNBC [33]. When administered as monotherapy, cetuximab appeared to have little activity in this setting. However, when combined with carboplatin, patient outcomes were enhanced with cetuximab (objective response 18 % and CB 27 %), although efficacy was short-lived [34]. Significant AEs hinder the combination of cetuximab with irinotecan and carboplatin [35], although promising findings were obtained in a study of cetuximab in combination with cisplatin in patients with TNBC, resulting in a significant PFS improvement compared to cisplatin alone [12]. Although the results in the present study show a tolerable safety profile and moderate activity in a limited number of patients for afatinib as monotherapy, it remains to be seen whether efficacy may be enhanced in this setting when combined with chemotherapy.

The role of other molecular targets in TNBC is also under investigation. Iniparib, in combination with gemcitabine and carboplatin, failed to improve improved OS compared with gemcitabine and carboplatin alone in a phase III study in patients with TNBC [36]. Results of an ongoing study of combination treatment with the mammalian target of rapamycin inhibitor, temsirolimus, and the irreversible EGFR/HER2 TKI, neratinib, in patients with metastatic HER2-amplified or TNBC (Clinical Trials.gov, study number: NCT01111825) are eagerly awaited. Indeed, improved molecular characterization of subtypes such as TNBC may enhance the likelihood of treatment success, enabling therapy to be tailored from an ineffective regimen at an earlier stage [2]. With this in mind, samples from patients enrolled in the cetuximab trials have been studied to identify potential predictive biomarkers [37]. Preliminary results suggest that cetuximab benefit may be correlated with lower expression of alpha-B-crystallin, higher expression of PTEN, and EGFR expression in basal TNBC [37]. More research into the biological properties and treatment options for patients with TNBC is clearly needed.

The PK findings in this study were comparable to the data observed in previous phase I studies of afatinib [38–41]. As expected, there were no obvious differences in plasma concentrations between patients in Cohort A and Cohort B. There was no indication of a systemic increase or decrease in afatinib trough values up to 6 months of treatment with afatinib (data not shown). The overall variability was moderate to high, which may have been caused by the various concomitant medications taken by the patients and also a possible influence of previous anti-cancer therapies.

## Conclusions

This study demonstrates that afatinib achieved CB for at least 4 months in a small number of heavily pretreated unselected patients with TNBC. Diarrhea and skin rash were the most commonly reported treatment-related AEs; diarrhea led to dose reductions in a large proportion of patients. Early implementation of concomitant treatment for managing AEs and lowering the starting dose of afatinib to 40 mg daily, as has been done in all ongoing phase II and phase III trials of afatinib in breast cancer, may enhance the ability of patients to adhere to treatment and thus improve outcome. Given the low number of patients that derived benefit from treatment in this trial, further examination of afatinib in patients with TNBC will require the identification of a selected population with ErbB network deregulation hallmarks, such as EGFR or HER2 mutations, ErbB receptor/ligand overexpression or activation, to increase the likelihood of a meaningful CB from an EGFR/HER2-targeting therapy.

## Electronic supplementary material

Below is the link to the electronic supplementary material.

Supplementary material 1 (DOCX 16 kb)
