# Supplementary material for: A phase II trial to assess efficacy and safety of afatinib in extensively pretreated patients with HER2-negative metastatic breast cancer
Source: Breast Cancer Res Treat. 2012 Jul 5;134(3):1149–59. doi: 10.1007/s10549-012-2126-1 (PMC3409367; doi:10.1007/s10549-012-2126-1)
Supplement: Supplementary file 1 — Supplementary material 1 (DOCX 16 kb) [file 10549_2012_2126_MOESM1_ESM.docx]

Supplementary biomarker methodology

Tissue slides from formalin fixed, paraffin embedded tissue were generated at 4 μm. HER2 IHC was performed with the HercepTest (DAKO) using the DAKO Autostainer Plus according to the manufacturer’s instructions. An oil-bath was used for antigen retrieval. A score of 3 + was defined as HER2-positive; IHC scores of 2 + were investigated using FISH. The pharmDx kit (DAKO) was used according to the manufacturer’s instructions. In cases where the HER2/CEP17 ratio was between 1.8 and 2.2, the FISH reading was repeated with a 40 cell count. A HER2/CEP17 ratio ≥ 2.0 was defined as HER2-positive. EGFR IHC was performed with the pharmDx test (DAKO) using the DAKO Autostainer Plus according to the manufacturer’s instructions. EGFR-positive staining was defined as any IHC staining of tumor cell membranes above background level irrespective of the completeness of membrane staining. ER and PgR testing was performed using the pharmDx test (DAKO) on the DAKO Autostainer Plus according to the manufacturer’s instructions. ER/PgR IHC staining was evaluated according to the Allred Scoring guideline described in the pharmDx kit; a total score of ≥ 3 was defined as positive. CK 5/14 IHC was performed with antibody clones D5/16/B4, M7237 (DAKO) using an autostainer and with mouse monoclonal antibody cocktail Mob 433 (Zytomed) on an autostainer. CK 5/6/14 evaluation was performed according to Nielsen et al. [1]. CK staining was scored positive if any cytoplasmic and/or membranous invasive carcinoma cell staining was observed. For analytical determination of serum HER2 ECD, EGFR ECD and CA 15.3 levels serum samples were collected on day 1 and 14 of courses 1 and 2, and day 1 of all subsequent courses and at end of trial, all prior to drug administration. Quantitative determination of circulating HER2 EDC was performed by ELISA (Siemens) according to the manufacturer’s instructions.

Reference

1. Nielsen TO, Hsu FD, Jensen K, Cheang M, Karaca G, Hu Z, Hernandez-Boussard T, Livasy C, Cowan D, Dressler L, Akslen LA, Ragaz J, Gown AM, Gilks CB, van de Rijn M, Perou CM (2004) Immunohistochemical and clinical characterization of the basal-like subtype of invasive breast carcinoma. Clin Cancer Res 10 (16):5367-5374. doi:10.1158/1078-0432.CCR-04-022010/16/5367 [pii]
